# Vertical Transmission of *Listeria monocytogenes*: Probing the Balance between Protection from Pathogens and Fetal Tolerance

**DOI:** 10.3390/pathogens7020052

**Published:** 2018-05-25

**Authors:** Nicole M. Lamond, Nancy E. Freitag

**Affiliations:** Department of Microbiology and Immunology, University of Illinois at Chicago, Chicago, IL 60612, USA; nlamond2@uic.edu

**Keywords:** pregnancy, vertical transmission, placenta, immunology, model systems

## Abstract

Protection of the developing fetus from pathogens is one of the many critical roles of the placenta. *Listeria monocytogenes* is one of a select number of pathogens that can cross the placental barrier and cause significant harm to the fetus, leading to spontaneous abortion, stillbirth, preterm labor, and disseminated neonate infection despite antibiotic treatment. Such severe outcomes serve to highlight the importance of understanding how *L. monocytogenes* mediates infiltration of the placental barrier. Here, we review what is currently known regarding vertical transmission of *L. monocytogenes* as a result of cell culture and animal models of infection. In vitro cell culture and organ models have been useful for the identification of *L. monocytogenes* virulence factors that contribute to placental invasion. Examples include members of the Internalin family of bacterial surface proteins such as Interalin (Inl)A, InlB, and InlP that promote invasion of cells at the maternal-fetal interface. A number of animal models have been used to interrogate *L. monocytogenes* vertical transmission, including mice, guinea pigs, gerbils, and non-human primates; each of these models has advantages while still not providing a comprehensive understanding of *L. monocytogenes* invasion of the human placenta and/or fetus. These models do, however, allow for the molecular investigation of the balance between fetal tolerance and immune protection from *L. monocytogenes* during pregnancy.

## 1. Introduction

During pregnancy, the placenta is both guardian and gate-keeper to protect and nurture the developing fetus. In addition to enabling the exchange of nutrients and waste, the placenta plays a critical role in protecting the developing fetus from potentially harmful pathogenic organisms. The guardian role of the placenta is largely successful during pregnancy, however, there are a few pathogens that are able to successfully circumvent the barrier functions and invade and multiply within the fetus [1]. These pathogens cause devastating effects, often leading to abortion or serious injury or death of the newborn. As a rule, pathogens capable of subversion of the placenta and infection of the fetus exhibit at least partial intracellular life cycles [1,2]. *Listeria monocytogenes* is one of these select pathogens that successfully targets and multiplies within the cells of the placenta and fetus.

*L. monocytogenes* is best known for being a gram-positive food-borne facultative intracellular bacterium that generally causes limited gastroenteritis in healthy individuals while causing severe invasive disease in immunocompromised populations [3,4,5]. The bacterium is widespread in the environment, living as a saprophyte in decaying plant matter, soil, and water. While *L. monocytogenes* does not form spores, it is able to survive numerous environmental stresses including low temperatures, changes in pH, high osmolarity, and exposure to metal ions [6,7]. *L. monocytogenes* transitions from an environmental bacterium to a foodborne pathogen following the consumption of contaminated food by a susceptible host [6,7,8,9,10,11]. *L. monocytogenes’* ability to withstand a variety of stress conditions contributes to the organism’s ability to contaminate and survive within food processing facilities, resulting in numerous recurring food recalls often linked to illness and death [12,13,14,15,16].

As mentioned above, *L. monocytogenes* infection in healthy individuals generally results in mild gastroenteritis, however, individuals that are immunocompromised, including the elderly, can develop severe invasive disease that manifests as meningitis, meningoencephalitis, and brain abscesses [17]. Severe invasive disease is associated with a high mortality rate of approximately 20% despite antibiotic treatment [17]. Pregnant women are also more susceptible to developing listeriosis and are ten times more likely to become infected than non-pregnant healthy individuals [18]. *L. monocytogenes* crossing of the maternofetal barrier leads to spontaneous abortion, stillbirth, preterm labor, and disseminated fetal infection with fetal and neonate death occurring in about 20–60% of reported cases [18,19,20]. *L. monocytogenes* can cause infection any time during pregnancy, but is most often diagnosed in the third trimester [20]. Maternal infection can present as asymptomatic or flu-like symptoms making diagnosis difficult; this situation likely contributes to late diagnosis and adverse outcomes for the fetus [20]. Given the burden of susceptibility to *L. monocytogenes* infection in pregnant women and the poor prognosis following infection, it is critical to better understand the mechanisms that enable *L. monocytogenes* to cross the placenta and infect the fetus. This review will explore the most recent knowledge regarding *L. monocytogenes* vertical transmission, compare the currently available animal models as well as cell culture models of infection, and will include a brief summary of what is currently known regarding maternal defenses against *L. monocytogenes* invasion.

## 2. Structural and Physiological Comparison of Animal Models Used to Assess *L. monocytogenes* Vertical Transmission

**Figure 1:**
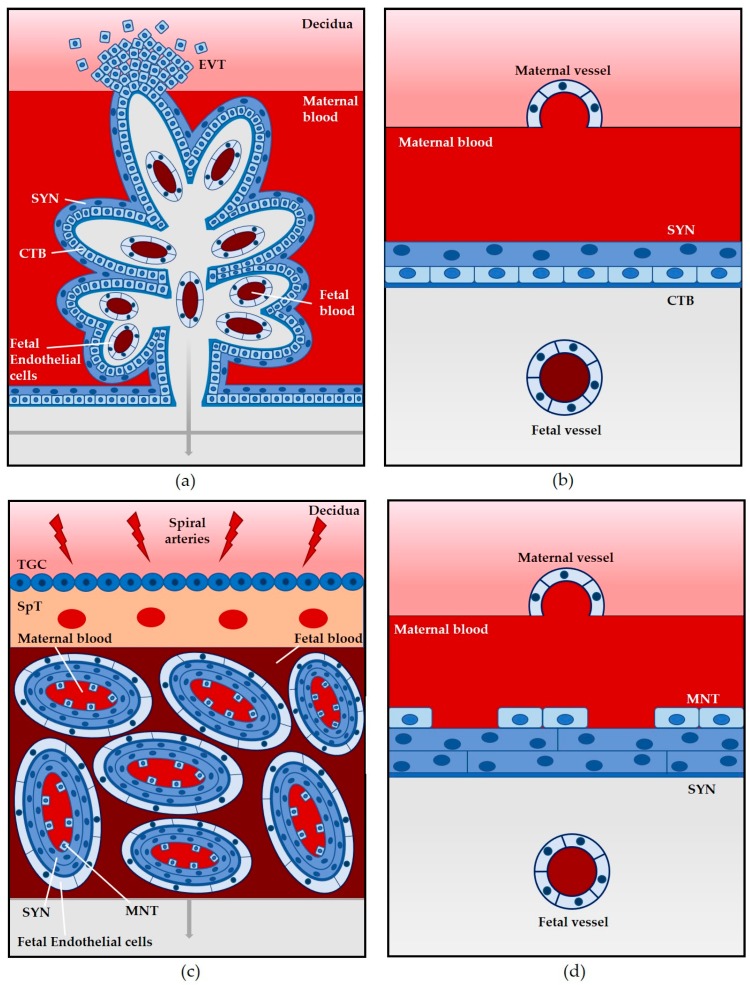
Direct comparison of placental structures. Humans, mice, guinea pigs, gerbils, and non-human primates all have a hemochorial placenta in which maternal blood comes into direct contact with fetal trophoblast cells. (**a**) Hemomonochorial villous placenta. Floating villi are surrounded by maternal blood with anchoring villi attached to the decidua with extravillous cytotrophoblasts (EVT) invading the decidua. An outer layer of syncytiotrophoblasts (SYN), a layer of cytotrophoblasts (CTB), and a layer of fetal endothelial cells create a barrier between the maternal and fetal blood; (**b**) Hemomonochorial placental barrier. A single layer of SYN in direct contact with maternal blood, a layer of CTB, and fetal endothelial cells constitute the placental barrier; (**c**) Hemotrichorial labyrinth placenta. Maternal and fetal blood are separated by two layers of SYN and a discontinuous layer of mononuclear trophoblasts (MNT). Trophoblast giant cells (TGC) and a spongiotrophoblast (SpT) region anchor the labyrinth to the decidua; (**d**) Hemotrichorial placental barrier. A discontinuous layer of MNT and a layer of SYN are in direct contact with maternal blood. A second layer of SYN and fetal endothelial cells complete the barrier. Modified and adapted from Maltepe et al. and Moffett et al. [23,30].

In addition to providing the fetus with nourishment and removing waste material during development, the placenta has evolved important barrier functions to prevent pathogens from infecting the fetus, while maintaining fetal tolerance in the mother [1,21]. The placenta consists of both maternal and fetal derived cells [1,22,23]. The cellular architecture of the placenta varies among mammals, with the human placenta consisting of a branching villi structure that includes both floating villi and villi that are anchored into the decidua or uterine lining (Figure 1a). It is hemochorial in that the maternal blood comes into direct contact with specialized fetal derived trophoblast cells that line the floating villi (Figure 1a,b) [1,22]. There is a continuous layer of fused multinucleated syncytiotrophoblasts in direct contact with maternal blood; these cells have differentiated and formed syncytia from the underlying cytotrophoblast cells (Figure 1a,b). The villous stroma separates the cytotrophoblast cells from the fetal blood. Some cytotrophoblast cells invade the decidua to form anchoring villi (extravillous cytotrophoblast cells) (Figure 1a) [21,22].

Available animal models for the study of *L. monocytogenes’* vertical transmission have structural and cellular differences in comparison to the human placenta. Animal models are critical, however, for understanding how *L. monocytogenes* gains access to placental cells and fetal tissues during pregnancy, a complex physiological state that cannot be replicated in cell or organ culture. To date, mice, guinea pigs, gerbils, and non-human primates have been utilized to study *L. monocytogenes* infection of the placenta and fetus [24,25,26,27,28,29]. We will contrast these models below.

### 2.1. Mouse

The mouse is one of the most commonly used and most economical laboratory mammals. One key advantage of the mouse as a model for infection has been the widespread availability of transgenic mouse lines and the diversity of genetic mutants available [24]. The gestation period for mice is only three weeks, and they have large litters and thus provide multiple opportunities to investigate fetal infection within a single animal; however, mice deliver altricial young in which many aspects of fetal development that occur in utero in humans occurs post-natally in mice [24].

Similar to humans, the mouse placenta is hemochorial [27]. There are, however, a number of differences in mouse placental structure in comparison to humans. The mouse placenta exhibits a labyrinth pattern and has three trophoblast layers: two syncytiotrophoblast cell layers that are adjacent to fetal endothelial cells and one non-continuous layer of mononuclear trophoblasts that is outside of the syncytiotrophoblast layer and in contact with maternal blood (Figure 1c,d) [24,27,31]. Additionally, the trophoblast invasion into the decidua is shallower in mice than humans [24].

One aspect of *L. monocytogenes* infection that merits consideration when using the mouse as a model of infection is the apparent species specificity of the bacteria for the invasion of some mouse cell types. *L. monocytogenes* expresses a variety of surface proteins that contribute to host cell invasion, and the Interalin (Inl)A protein has been shown to be important for bacterial invasion of host intestinal epithelial cells through the targeting the host E-cadherin receptor [3,7]. In mice, there is a single amino acid variation in the E-cadherin receptor such that a proline in humans is replaced by a glutamate in mice, significantly impairing the interaction of *L. monocytogenes* InlA with its receptor in mice [26,29,32,33]. Two methodologies have been developed that could facilitate the understanding of InlA-dependent invasion in mouse models of *L. monocytogenes* vertical transmission: the selection of bacterial InlA mutants with enhanced affinity for mouse E-cadherin receptor (murinization of InlA) and the contrasting humanization of the mouse E-cadherin receptor through the expression of human E-cadherin in mice. Each approach has strengths and weaknesses: the mouse optimized InlA (InlAm) binds mouse E-cadherin with high affinity, however, InlAm has also been reported to exhibit increased binding affinity for other cadherins such as N-cadherin, thereby potentially altering *L. monocytogenes* cell tropism [34,35]. Humanization of the mouse E-cadherin has been achieved through the use of transgenic lines [26,29]. It is possible that the altered E-cadherin may exhibit changes in affinity for its other host cell binding partners.

Another aspect of the mouse model to consider is the route of infection: oral vs intravenous. *L. monocytogenes* infection typically occurs via the consumption of contaminated food. Using the oral route of infection in the mouse model is more representative of the typical exposure to *L. monocytogenes* infection, however, this route of infection in the mouse model requires a high infection dose and can lead to highly variable colony forming unit (CFU) counts in the intestine, liver, and spleen [26,36]. Alternatively, the more widely used intravenous route of infection bypasses the crossing of the gastrointestinal barrier by injecting bacteria directly into the bloodstream and has been shown to lead to highly reproducible data, although a caveat is the likely artificial large bolus of bacteria that immediately reach the liver and spleen from the bloodstream [26].

Despite these limitations, the mouse has thus far served as the most accessible model for the study of *L. monocytogenes’* vertical transmission, facilitating the identification of both host and bacterial factors that contribute to infection [27,37,38,39,40,41,42].

### 2.2. Guinea Pig

Another useful animal model to study *L. monocytogenes* placental infection is the guinea pig. Similar to humans, guinea pigs have a long gestation resulting in precocial or well-developed, young [24]. While the animal is a natural host for *L. monocytogenes*, the course of *L. monocytogenes* infection in the guinea pig appears to differ from humans in that invading bacteria do not exhibit strong central nervous system tropism in the guinea pig [26]. However, bacteria infecting guinea pigs do exhibit placental tropism. Like other rodents, guinea pig placentas have a labyrinth pattern, as opposed to the villous structure seen in humans [43]. Similar to humans, the placenta is hemochorial with a single layer of syncytiotrophoblasts in direct contact with the maternal blood. Also similar to humans, there is invasion of the decidua by extravillous cytotrophoblasts [24,25]. Guinea pigs do not have the advantages of mice in that they are genetically variable, and transgenic animals have not routinely been generated, however, with the advent of CRISPR technology, that situation may ultimately change.

Similar to the situation with the mouse E-cadherin receptor, *L. monocytogenes* host cell tropism may be altered during pregnancy in that the bacterial surface protein InlB that contributes to bacterial invasion of multiple cell types does not bind with high affinity to its Met and gC1q-R receptors in the guinea pig [26]. Transfection experiments in which human Met and gC1q-R were transfected into guinea pig cells demonstrated a gain in function in these cells for InlB binding [26,44], however, unlike the mouse, receptor gene knock-in animals have yet to be constructed and bacterial InlB mutants with enhanced affinity for guinea pig receptor have thus far not been isolated.

### 2.3. Gerbil

Gerbils are naturally susceptible to *L. monocytogenes* infection [29]. Similar to other rodents, the gerbil placenta exhibits a labyrinth pattern. The placenta is hemochorial and, as in mice, there are three layers of trophoblast cells [45]. One attractive aspect of the gerbil model that sets it apart from the mouse and the guinea pig is that both *L. monocytogenes* InlA and InlB are able to interact with and bind to their respective receptors, making this model a feasible alternative to the mouse and guinea pig for studying internalin-dependent invasion [29,46].

### 2.4. Non-Human Primates

Of all of the available animal models, the placental structure of non-human primates, specifically old world monkeys such as macaques and baboons, most closely resembles that of humans as these animals are most closely related to humans [24]. The placenta is villous, hemochorial, and extravillous cytotrophoblasts invade into the maternal decidua [24,28]. One difference between the human placenta and the placentas of old world monkeys is that cytotrophoblasts that spread out from the anchoring villi form a continuous, slightly thicker trophoblast shell that is delineated from the endometrium, and there is an absence of interstitial trophoblasts or trophoblasts that invade the decidua and surround but do not invade spiral arteries [21,24]. In contrast with old world monkeys, human extravillous cytotrophoblasts exhibit both interstitial and endovascular invasion [24]. While structurally most similar, the cost and long gestational period of non-human primates has limited the number of *L. monocytogenes* vertical transmission studies conducted in these animals.

## 3. Lessons from Available Animal Models: Routes of Placental Entry and Bacterial Factors that Contribute to *L. monocytogenes* Vertical Transmission

**Figure 2:**
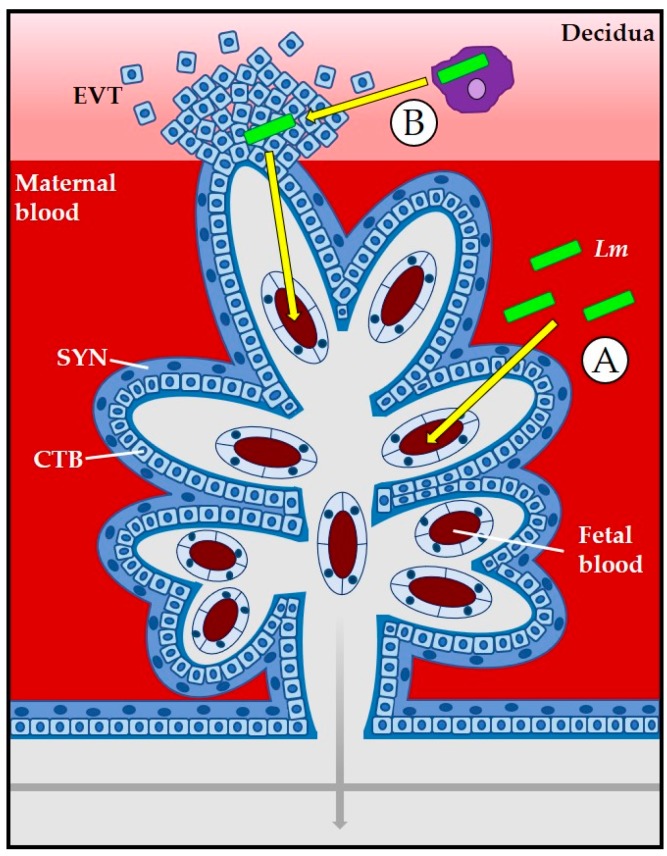
At the placental barrier, *L. monocytogenes* (*Lm*, green rods) can invade the placenta via direct invasion of syncytiotrophoblasts (SYN) (**A**) or through cell-to-cell spread from the decidua or from bacteria located within maternal leukocytes to extravillous cytotrophoblasts (EVT) (**B**). Modified and adapted from Robbins and Bakardjiev [1].

*L. monocytogenes* exhibits multiple tissue tropisms during host infection, including the targeting of the placenta and fetus. Two routes of mammalian cell entry that are available to *L. monocytogenes* are direct bacterial invasion of cells through interactions of cell receptors with bacterial surface proteins and the invasion of a cell as a result of bacterial spread through the cytosol of an adjacent cell (cell-to-cell spread). Syncytiotrophoblasts are placental cells that are in direct contact with maternal blood and thus they offer *L. monocytogenes* a portal of direct entry dependent on bacterial proteins binding to host cell surface receptors (Figure 2). Following receptor-mediated cell entry, *L. monocytogenes* escapes from a membrane-bound vacuole through the secretion of the pore forming toxin listeriolysin O (LLO) as well as two phospholipases (phosphatidylinositol-specific phospholipase C, PI-PLC, and a broad specificity phosphatidylcholine phospholipase C, PC-PLC) to enter the cytosol where bacterial replication occurs. The bacterial surface protein ActA stimulates host cell actin polymerization and provides *L. monocytogenes* with a motile force to move through the cytosol and eventually force entry into adjacent neighboring cells. *L. monocytogenes* uses cell-to-cell spread to travel through placental cells to reach the fetus [3,7,11,47]. Additional routes of *L. monocytogenes* entry into the placenta include the invasion of extravillous cytotrophoblasts via cell-to-cell spread from the maternal decidua or through bacterial trafficking within maternal immune cells (Figure 2) [1,22]. Placental infection is required for fetal infection as *L. monocytogenes* must travel through layers of placental cells prior to encountering the fetus [27].

### 3.1. The Placenta as a Barrier to L. monocytogenes Infection

*L. monocytogenes* can invade the placenta via extravillous cytotrophoblasts that have anchored into the decidua. It has been shown that these cells are susceptible to *L. monocytogenes* infection [22], however, intracellular growth and bacterial spread from these cells into trophoblasts deeper into the placenta has been shown to be impaired [48]. The replication defect of *L. monocytogenes* within these cells may reflect an impairment in bacterial escape from the vacuole, leading to degradation of invading bacteria in lysosomes [48,49].

Syncytiotrophoblasts comprise the outer-most layer of villous trophoblast cells in humans and as such are in direct contact with maternal blood. Syncytiotrophoblasts are fused multinucleated cells and do not have intercellular junctions. Given that many pathogens use receptors present within intercellular junctions as entry points during infection, including *L. monocytogenes* [49], the absence of these junctions and their affiliated receptors may constitute an effective defense against pathogen entry and/or translocation to underlying cells. Additionally, Zeldovich et al. showed that syncytiotrophoblast cells have a higher elastic modulus than murine trophoblast stem cells (mTSCs) (undifferentiated trophoblast cells which show similarities to mononuclear trophoblasts), potentially due to the unique actin cytoskeleton structure in these cells that contributes to increased structural rigidity [48]. If this surface resistance is disrupted, susceptibility to *L. monocytogenes* is increased [48].

### 3.2. Role of Bacterial Internalin Proteins in Placental and Fetal Invasion

As mentioned briefly above, internalins are a family of *L. monocytogenes*-secreted and surface proteins that contribute to direct bacterial invasion of host cells. Three internalins have been implicated in *L. monocytogenes* infection of the placenta in animal and cell culture models: InlA, InlB, and the recently identified InlP.

Placental syncytiotrophoblast cells express both E-cadherin and Met, the receptors for InlA and InlB, respectively [38]. Both of these internalins have been implicated in the direct invasion of syncytiotrophoblasts in cell and organ culture. Lecuit et al. demonstrated that InlA mediates *L. monocytogenes* invasion of BeWo cells (a humanchoriocarcinoma cell line representative of syncytiotrophoblasts), primary human trophoblast cells, and human placental explants [43]. Bakardjiev et al. confirmed the importance of InlA in the infection of BeWo cells and primary human trophoblasts [25]. There exists some controversy, however, as to the role of InlA in placental invasion as it has been observed that InlA contributed no apparent role in the invasion of the placenta when using the pregnant guinea pig model, an animal with the correct form of the E-cadherin receptor normally bound by InlA [25]. E-cadherin has also been reported to be absent on the apical surface of syncytiotrophoblasts [22,49].

Gessain et al. demonstrated a critical role for InlB in the invasion of syncytiotrophoblasts through the comparison of *L. monocytogenes* infection of human intestinal LS174T cells and human trophoblast Jar cells. Binding of InlB to the Met receptor results in the activation of the phosphoinositide 3-kinase (PI3-K) signaling cascade [38,50,51,52]. In tissues in which PI3-K signaling is constitutively active, such as the intestine, InlB does not appear necessary for invasion; however, in tissues such as the placenta, PI3-K signaling is not constitutively active and InlB is necessary to activate PI3-K to enable *L. monocytogenes* entry into syncytiotrophoblast cells [38]. However, similar to InlA, the significance of InlB in placental invasion has been debated. Bakardjiev et al. and Robbins et al. found no significant difference between *L. monocytogenes* 10403S mutants lacking InlB and wildtype bacteria in BeWo cells, primary human trophoblasts, human placental explants, and in vertical transmission within the guinea pig model (which lacks a high affinity InlB receptor) [22,25].

Indeed, controversy exists regarding the ability of *L. monocytogenes* to directly invade syncytiotrophoblasts. Using human placental explants, Robbins et al. found that *L. monocytogenes* uses InlA to target and invade extravillous cytotrophoblasts that invade into the maternal decidua rather than syncytiotrophoblasts [22]. It is possible that differences observed in *L. monocytogenes* syncytiotrophoblast invasion reflect the use of placental explants derived from first and third trimester placentas by Robbins et al. and Gessain et al., respectively. The placenta is not fully developed until the end of the first trimester of pregnancy when maternal blood begins to flow from spiral arteries and fill the intervillous space [21]. Furthermore, the villous blood vessels continue to grow through the beginning of the third trimester as the fetus grows and metabolic demands are increased [21]. The state of placental development could influence the efficiency of *L. monocytogenes* cell entry.

Recently, a novel virulence factor, InlP was identified by Faralla et al. through the use of a transposon insertion mutant screen in pregnant guinea pigs [53]. InlP was found to confer a strong placental invasion tropism in both mice and guinea pigs. Loss of InlP was found to impair bacterial intracellular growth and/or cell-to-cell spread in human placental explants [53]. It has been hypothesized that *L. monocytogenes* must be able to spread from extravillous cytotrophoblasts in order for transmission to the fetus to occur, and that these cells may be able to restrict intracellular growth and the spread of *L. monocytogenes* [54]. The work by Faralla et al. using human trophoblast progenitor cells (hTPCs) as a cellular model demonstrated that InlP contributes to overcoming this barrier [53].

### 3.3. Role of LLO and Phospholipases

Once *L. monocytogenes* successfully invades a host cell, it resides within a membrane-bound vacuole. The secretion of LLO and two bacterial phospholipases facilitates the formation of vacuolar membrane pores and membrane dissolution, enabling *L. monocytogenes* to escape into the cytosol where bacterial replication can occur. Bacterial mutants lacking LLO are defective for vacuole escape and bacterial replication and are highly attenuated in animal infection models [55]. Perhaps not surprisingly, Monnier et al. found that while *L. monocytogenes* mutants lacking LLO were capable of low level of invasion of the placenta, they were unable to infect the fetus, a further indication of the necessity of placental invasion prior to fetal invasion [56].

### 3.4. Role of ActA

As described above, one portal of *L. monocytogenes* cell entry is via cell-to-cell spread, a process dependent upon bacterial expression of ActA [3,7,11,47]. Given that the placental barrier is made up of multiple layers of cells that separate the maternal and fetal blood, intracellular pathogens that are able to spread from cell to cell, such as *L. monocytogenes*, have an advantage in being able to cross the placental barrier and infect the fetus. Bakardjiev et al. used *L. monocytogenes* mutants lacking ActA to demonstrate that bacterial cell-to-cell spread is critical for infection of the fetus in guinea pigs [57]. Monnier et al. further demonstrated the importance of ActA in infection of the fetus in mice [56]. Overall, these studies strongly suggest that fetal infection only occurs as a result of direct spread of bacteria from infected placental cells.

## 4. Balancing Fetal Tolerance with Protection against Pathogens: Maternal Immune Responses to *L. monocytogenes*

Pregnancy necessitates a unique situation in which the fetus must be protected from the maternal immune responses against non-self while still maintaining protection of the mother and fetus against pathogens. This complicated balance between fetal rejection and susceptibility to infection is orchestrated via the regulation of maternal immune effector cells. The immune cells present at the maternofetal barrier consist of cells that reside in the decidua [58]. Natural killer (NK) cells comprise a majority of the immune cell population (~70%). Macrophages make up approximately 20% of the population with the T cells ranging from 10–20% [58,59].

### 4.1. Cells and Cell Signaling at the Maternofetal Interface

The NK cells of the decidua consist of a subset of NK cells known as the decidual NK (dNK) cells, and these cells have been shown to be important during early pregnancy. The dNK cells have been shown to remodel spiral arteries to ensure increased maternal blood flow through the placenta as well as regulate extravillous cytotrophoblast invasion of the decidua and spiral arteries [30,58]. Disruption of this interaction between dNK cells and trophoblasts can lead to pregnancy complications such as preeclampsia [30,58]. It has been suggested that dNK cells produce interferon gamma IFNγ during the process of remodeling and that IFNγ acts on non-dNK cells, which may contribute to the interaction with and regulation of trophoblast cells [58]. The dNK cells also produce the immunosuppressive cytokine IL-10 that may lead to differentiation of decidual macrophages and allow for this subset of macrophages to be maintained in a noninflammatory state [58]. Additionally, there is evidence that decidual macrophages may play a role in the remodeling process by clearing cell debris and apoptotic cells [58].

Trophoblast cells have developed mechanisms to avoid detection by the maternal immune response. Syncytiotrophoblast cells do not express MHC class I or class II molecules, allowing them to remain undetected by maternal T cells with the αβ receptor [30,60]. This form of placental evasion of the maternal immune response preserves placental cells but also means that infected cells will not be recognized by cytotoxic T lymphocytes. While there is some evidence that extravillous cytotrophoblast cells express HLA-A, HLA-B, and HLA-C early during pregnancy, extravillous cytotrophoblast cells that invade the decidua typically express HLA-C, HLA-G, and HLA-E, but not HLA-A or HLA-B, which would initiate a maternal immune response [30,61]. HLA-G may act as a signal for pregnancy related functions [30]. Additionally, maternal Foxp3^+^ T regulatory cells (Tregs), T cells associated with immune suppression, are important for maintaining fetal tolerance during pregnancy [42,62].

The appropriate regulation of cytokine signaling is also critical for a successful pregnancy. Increased expression of Th1 cytokines such as IFNγ, TNFα, and IL-2 can result in detrimental outcomes for pregnancy [40,60,63]. Th2 cytokines have been identified at the maternofetal interface and may indicate an environment that is more tolerable to the fetus [63,64].

As might be anticipated, the various stages of pregnancy (implantation/placentation, fetal development, and labor and delivery) translate into a complex and perhaps ever-changing balance between fetal tolerance and maternal immunity. Mor and Cardenas proposed that the multiple stages of pregnancy induce variations in the maternal immune response from pro-inflammatory to anti-inflammatory back to pro-inflammatory for implantation/placentation, fetal development, and labor, respectively [59]. Taken together, many challenges exist in attempting to fully comprehend the complexity of immune regulation during pregnancy and how protection against pathogens may be compromised during specific stages of pregnancy [59,65].

### 4.2. Maternal Immune Responses to L. monocytogenes during Pregnancy

With the unique immunological environment exhibited during pregnancy, the host must attempt to combat *L. monocytogenes* infection while maintaining fetal tolerance and survival. A robust innate immune response is critical for protection against *L. monocytogenes* invasion of the placenta and fetus. Neutrophils are recruited to sites of infection by cytokines such as IL-6 and, in turn, secrete chemokines such as colony stimulating factor-1 (CSF-1) and MCP-1 to recruit macrophages [66]. Macrophages secrete TNFα and IL-12, which signal NK cells to produce IFNγ, leading to activation of macrophages and an increase in the efficacy of their bactericidal response [66]. These facets of innate immunity have been shown to be critical for the initial control of *L. monocytogenes* [66,67,68,69]. Following the initial innate immune response to *L. monocytogenes*, the host must be able to produce an effective adaptive immune response to clear the infection. Activated dendritic cells (DC) are able to prime T cells, which are critical for the clearance of *L. monocytogenes* [66,67]. As *L. monocytogenes* is an intracellular bacterium, CD4 and CD8 T cells are the primary adaptive immune response [66,67]. A successful battle results in clearance of the infection and a healthy baby. However, common outcomes of *L. monocytogenes* infection during pregnancy include spontaneous abortion, still birth, and preterm labor, indicating a failure to control infection without harm to the developing fetus. Specific aspects of maternal immunity relevant to *L. monocytogenes* infection as indicated through the study of animal models are briefly discussed below.

During pregnancy, colony stimulating factor-1 (CSF-1) is produced by the uterine epithelium in significant amounts and has been shown to play a role in placental development [70,71]. Trophoblast cells bear CSF-1 receptors and once CSF-1 is produced, it is able to target trophoblast cells. Of particular interest during infection with *L. monocytogenes*, CSF-1 targeting of trophoblasts induces production of neutrophil chemoattractants (KC) and macrophage inflammatory protein-2 (MIP-2) [41]. This in turn recruits neutrophils to the site of infection in the decidua. In addition to neutrophils, CSF-1 dependent macrophages are recruited to the decidua and their function is to combat *L. monocytogenes* in this location [72]. While it has been shown that there is an abundance of dNK in the decidua, these cells are not required during the immune response to *L. monocytogenes* in mice [73]. Additionally, initial infection of the decidua by *L. monocytogenes* appears to exhibit a bottleneck effect and may be limited due to cell-autonomous defense mechanisms of decidual stroma and/or endothelial cells or limited access of trafficked infected cells from the maternal blood to the decidua [74]. When *L. monocytogenes* overcomes this bottleneck and establishes infection, an impaired immune response during early stages of infection allows for *L. monocytogenes* to grow and spread within the placenta [74].

DCs are another cellular arsenal of the immune response that differentiate during pregnancy into a unique DC subset. Trophoblasts secrete pregnancy-specific glycoproteins (PSGs) which are essential to a successful pregnancy [75]. PSG1a is able to initiate differentiation of DCs into a specific subset of DCs that is unique to pregnancy and that secretes IL-6 and TGFβ [75]. DCs that were matured by PSG1a induce Foxp3^+^ Tregs and CD4^+^ T cells to produce Th2 cytokines and IL-17 [75]. Th1 cytokines levels, such as IFNγ, which have been shown to be important in *L. monocytogenes* clearance, are reduced during pregnancy, which may contribute to the susceptibility of pregnant women to *L. monocytogenes* infection [37].

Using a pregnant mouse model, Nancy et al. showed that during pregnancy, effector T cells fail to accumulate with decidual stroma cells due to epigenetic silencing of pro-inflammatory chemokine genes that are important for recruiting effector T cells [76]. This chemokine silencing allows for protection against inflammation at the maternal-fetal interface, thus protecting the fetus. Another layer of protection is conferred by expression of maternal Foxp3^+^ Tregs that contribute to the maintenance of pregnancy and fetal tolerance; however, an unfortunate caveat is that the immune suppression mediated by Tregs leaves the placenta vulnerable to intracellular pathogens such as *L. monocytogenes* [42,62]. Interestingly, *L monocytogenes* does not have to invade the placenta to cause fetal injury. Studies by Rowe et al. [62] using mice bearing allogenic pregnancies to mimic the natural genetic heterogeneity that occurs between mother and fetus indicate that *L. monocytogenes* infection can result in ‘sterile’ fetal wastage, where fetal injury occurs as a result of infection-induced inflammation, and that this phenomenon does not appear to require direct bacterial invasion in utero. Instead, *L. monocytogenes* infection overrides the suppression mediated by maternal Tregs and stimulates the expansion and IFN-γ production by maternal T cells with fetal specificity [62,77]. More recent studies by Chaturvedi et al. [78] indicate that the fetal wastage stimulated by *L. monocytogenes* infection results from the placental recruitment of CXCL9-producing inflammatory neutrophils and macrophages that lead to the infiltration of fetal-specific T cells into the decidua. Fetal-specific maternal CD8+ T cells were found to upregulate the expression of the chemokine receptor CXCR3 and to function together with neutrophils and macrophages to induce fetal resorption. Blockage of CXCR3 protected against fetal wastage and protected against the accumulation of maternal T cells with fetal specificity [78]. Taken together, it appears that *L. monocytogenes* promotes the pathogenesis of fetal infection by functionally overriding chemokine silencing at the maternal-fetal interface.

## 5. Summary

Pregnancy creates a unique environment in which a balance between fetal tolerance and pathogen protection must be achieved. The placenta serves as a barrier to protect the fetus; however, some pathogens, including *L. monocytogenes*, can cross this barrier and infect the fetus.

Mouse, guinea pig, gerbil, and non-human primate animal models are available to examine vertical transmission of *L. monocytogenes*; however, none of these models provide a complete representation of the placenta and vertical transmission of humans. Human placental cell lines and human placental explants provide avenues to explore invasion of these tissues, but do not provide a complete representation of an in vivo model, especially in regards to immunity.

Several *L. monocytogenes* virulence factors have been implicated in placental infection. Interalin (Inl) A, InlB, and InlP have been shown to contribute to the invasion of trophoblast cells; however, there has been controversy as to the roles of InlA and InlB in vertical transmission. This could be due in part to the limitations of the current animal models available. Other important virulence factors for placental infection and vertical transmission include LLO and ActA which are necessary for growth within placental cells and in cell-to-cell spread through the placenta to the fetus. Future studies should focus on clarifying the roles of internalins during placenta invasion and at placental infection sites.

The balance between fetal tolerance and pathogen protection must be maintained for successful pregnancy. The placenta acts as a barrier to pathogens, and maternal Tregs suppress the maternal immune system so that pregnancy can be sustained; however, this leaves the host more susceptible to pathogens that can cross the placental barrier such as *L. monocytogenes*. The complex placental environment changes throughout pregnancy and future studies should explore if the immune response to *L. monocytogenes* also exhibits changes through these stages. Additionally, the fetal immune system begins development in utero and it would be of interest to determine whether or not the immature, developing fetal immune response is capable of protecting the fetus from pathogens such as *L. monocytogenes*.
